# Brief screening questions for depression in chiropractic patients with low back pain: identification of potentially useful questions and test of their predictive capacity

**DOI:** 10.1186/2045-709X-22-4

**Published:** 2014-01-17

**Authors:** Alice Kongsted, Benedicte Aambakk, Sanne Bossen, Lise Hestbaek

**Affiliations:** 1 The Nordic Institute of Chiropractic and Clinical Biomechanics, Campusvej 55, 5230 Odense, M, Denmark; 2 Department of Sports Science and Clinical Biomechanics, University of Southern Denmark, Odense, Denmark

**Keywords:** Chiropractic, Depression, Questionnaires, Low back pain, Primary health care

## Abstract

**Background:**

Depression is an important prognostic factor in low back pain (LBP) that appears to be infrequent in chiropractic populations. Identification of depression in few patients would consequently implicate screening of many. It is therefore desirable to have brief screening tools for depression. The objective of this study was to investigate if one or two items from the Major Depression Inventory (MDI) could be a reasonable substitute for the complete scale.

**Methods:**

The MDI was completed by 925 patients consulting a chiropractor due to a new episode of LBP. Outcome measures were LBP intensity and activity limitation at 3-months and 12-months follow-up. Single items on the MDI that correlated strongest and explained most variance in the total score were tested for associations with outcome. Finally, the predictive capacity was compared between the total scale and the items that showed the strongest associations with outcome measures.

**Results:**

In this cohort 9% had signs of depression. The total MDI was significantly associated with outcome but explained very little of the variance in outcome. Four single items performed comparable to the total scale as prognostic factors. Items 1 and 3 explained the most variance in all outcome measures, and their predictive accuracies in terms of area under the curve were at least as high as for the categorised complete scale.

**Conclusions:**

Baseline depression measured by the MDI was associated with a worse outcome in chiropractic patients with LBP. A single item (no. 1 or 3) was a reasonable substitute for the entire scale when screening for depression as a prognostic factor.

## Introduction

Pain and depression often co-exist [1-3], and although the causal relation between the two is not clear [4,5], evidence suggests that pain negatively affects outcome in depression as well as vice versa [6].

Low back pain (LBP) is a highly frequent pain condition with a substantial impact on global health [7] for which the risk of a poor prognosis is increased in the presence of depression [8,9]. It is a condition for which there is no generally effective treatment, but non-pharmacological treatment addressing psychological symptoms in addition to the physical symptoms has been demonstrated to improve outcome in LBP patients with high scores on psychological questions [10].

Chiropractors see a large number of LBP patients who appear to be a population with relatively low frequency of severe psychological distress [11-13]. Nevertheless, a substantial proportion with borderline depressive scores has been observed [12]. Therefore, to improve care and to predict prognosis, it may be important to identify psychological factors, including depression, among those seeking care for LBP, also in chiropractic practice. For that purpose screening instruments generally perform better than clinical impressions [14,15], and a number of questionnaires screening for depression exist [16]. The Major Depression Inventory is thoroughly validated as a diagnostic screening instrument [17,18] and has been shown to be feasible in chiropractic care [13]. However, it is unknown if the MDI predicts outcome in chiropractic patients.

Despite the potential implications for management and prognosis, routine screening for psychological factors is not widely implemented [19] and has met some resistance from clinicians [20]. One reason might be that the questionnaires are too extensive for routine clinical use where information on a variety of other prognostic factors is also relevant to collect. This is especially true in chiropractic practice where the subpopulation with depressive symptoms is small [11-13], and therefore the identification of these relatively rare cases involves screening of many for whom it has no relevance.

If systematic screening for depression is to be implemented, there is a need for a very short and easily completed tool. Fair to good diagnostic accuracies of one- and two-item screening tools with full questionnaires for depression as reference standards have been demonstrated [21-23]. It is unknown whether observed variations are due to differences between screening questions or between screened populations, and it is possible that suitable screening questions differ between populations. Furthermore, the usefulness of brief screening questions for prediction of prognosis is unrevealed.

The aim of this study was to investigate whether the MDI is predictive of outcome in chiropractic practice and if so, whether one or two items from the MDI could potentially be used as an ultra-short tool for capturing depressive symptoms in LBP patients seeking chiropractic care. To obtain that we tested whether the total MDI was associated with 3- and 12-months outcomes, explored which single items of the MDI correlated best with the total score, and compared the predictive capacity of these items to that of the total MDI.

## Method

The study was incorporated in a previously described cohort study with data collection occurring at seventeen chiropractic clinics in the research network of the Nordic Institute for Chiropractic and Clinical Biomechanics in Denmark [24]. Patients completed questionnaires in the reception area prior to the first consultation due to a new episode of LBP. Follow-up questionnaires were mailed after 3 and 12 months. Treatment was unaffected by study participation and the chiropractors were free to choose the treatment they found appropriate. It was confirmed by The Regional Scientific Ethical Committees for Southern Denmark that the study did not need ethics approval according to Danish rules [25].

### Participants

Consecutive patients aged 18–65 years attending chiropractic practice for the first time due to their current episode of LBP, and who could read Danish were potential participants. Patients were not included if inflammatory or pathological pain were suspected, in case of nerve root involvement requiring acute referral to surgery, if pregnant, or if having had more than one health care consultation due to LBP within the previous 3 months. Patients were excluded if pathology was diagnosed as the reason for LBP during the course of the study.

### Measurements

Depression was measured by the MDI consisting of twelve items answered by choosing one of 6 response options from ‘At no time’ (= 0) to ‘All the time’ (= 5) [17] (Additional file 1). When using the MDI as a depression rating scale ten of the twelve items are used for the total score. Only the highest score on the items 8a and 8b is included since these are considered opposites (feeling restless and feeling slowed down) and similarly only the highest score on items 10a and 10b (reduced appetite and increased appetite) is included. This results in a sum score ranging from 0 to 50 that are categorised into ‘no depression’ (score of 0 – 19), ‘mild depression’ (score of 20 – 24), ‘moderate depression’ (score of 25 – 29), and ‘severe depression’ (score of 30 – 50) [18].

At baseline, patients also responded to questions regarding LBP duration (0–2 weeks, 2–4 weeks, 1–3 months, >3 months), number of previous episodes (0, 1–3, >3), LBP intensity (Numeric rating scale (NRS) 0–10 [26]), leg pain intensity (NRS 0–10), and activity limitation (Roland Morris Disability Questionnaire (RMDQ) proportional score 0–100 [27,28]).

Outcome measures were LBP intensity (NRS 0–10) and the RMDQ proportional score (0–100) after 3 and 12 months.

### Data analysis

Data were entered twice in EpiData [29]. Analyses were performed in STATA/SE 12.1 (STATA Corp, College Station, Texas, USA). No imputations were made, and sixteen subjects (1.7%) were excluded from the analyses due to missing values on one or more MDI items.

Associations between the MDI and outcome were investigated in linear regression models after categorisation of MDI since the sum score had a non-linear relation with outcome measures.

The identification of potential prognostic screening items was performed in two steps: (1) We identified the single items that best reflected the sum score of the scale by calculating the Spearman correlation between each item and the total score and the variance explained (R-squared) in a linear regression model with the MDI sum score as dependent variable and the single item as the independent variable. These were considered ‘candidate items’. (2) The associations between the candidate items and outcomes at 3-months and 12-months follow-up were then tested by means of linear regression, and the prognostic capacity was compared in terms of effect sizes (β-coefficients) and amount of variance in the outcome explained by the item (R-squared). Responses “Most of the time” and “All the time” were collapsed for this purpose due to few observations in these categories.

To report more clinically interpretable results the predictive abilities of the two preferred candidates, quantified as area under the ROC curve (AUC) and likelihood ratios (LH+/LH-), were compared to that of the total MDI when predicting ‘persistent pain’ (LBP intensity > 0) and ‘persistent activity limitation’ (RMDQ >8% corresponding to >2 points on the 24-item RMDQ [30]. For the total MDI, we considered all of mild, moderate, and severe depression as a positive test, since these categories were quite small. On the single items scores 2–5 were considered signs of depression based on ROC-curves and favouring a high sensitivity. Each single item was tested by itself, and the two best screening questions were combined defining a patient as depressed if scoring 2–5 on any of the two questions.

## Results

### Participants

The study cohort consisted of 925 patients (45% females, mean age 43 years) who had completed the MDI. Those excluded because of missing MDI items were 3.6 years older than participants, more often females, and their LBP had less frequently lasted for more than 4 weeks. No differences were observed on LBP severity or on the completed MDI items. Follow-up was completed by 731 (79%) and 684 (74%) after 3 and 12 months, respectively. Non-responders were more often males, were on average 5 years younger, and had slightly higher MDI scores (1.5 points) than those participating in follow-up. Patient characteristics are summarised in Table 1.

**Table 1:** Baseline characteristics of the cohort separated according to signs of depression

	**Total cohort n = 925**	**No depression n = 843**	**Mild to severe depression n = 82**	p-value*
Females	45%	43%	61%	<0.01
Age, mean (sd)	43 (12)	44 (12)	40 (12)	<0.01
Duration of LBP				<0.01
0-2 weeks	62%	65%	39%	
2-4 weeks	13%	13%	21%	
1-3 months	11%	11%	10%	
>3 months	14%	12%	30%	
>3 previous LBP episodes	49%	48%	57%	0.10
LBP intensity, median (IQR)	7 (5–8)	7 (5–8)	8 (6–8)	<0.01
Leg pain >0	56%	54%	72%	<0.01
RMDQ, mean (sd)	51 (24)	50 (24)	65 (20)	<0.01

*p-value for test of no difference between ‘no depression’ and ‘mild to severe depression’ the duration of LBP categories for non-depressed sum to 101% due to rounding of figures.

### MDI scores

Scores on the MDI were generally low (median 6, IQR 3–11) and 91% were categorised as non-depressed. The mild, moderate, and severe depression categories included respectively 4%, 3%, and 2% of participants. Single items were answered with “At no time” in 23% (Item 3: Lack of energy and strength) to 94% (Item 6: Life not worth living) of the cohort. Signs of depression were associated with female gender, younger age, and a more severe LBP profile (Table 1).

### Associations between the MDI sum score and outcome

The categorised MDI had statistically significant associations with LBP intensity and activity limitation at 3-months and 12-months follow-up (all p < .05). However, the effects, especially regarding pain outcomes, were small, and the amount of variance in the outcome explained by MDI was very low (Table 2).

**Table 2:** Associations between the total MDI scale and outcomes and between five candidate items and outcomes

	**β coefficients (95% CI)**			
	**LBP 3-months**	**LBP 12-months**	**RMDQ 3-months**	**RMDQ 12-months**
**Total MDI scale**				
MDI categorised	Adj. R^2^ = 0.03	Adj. R^2^ = 0.01	Adj. R^2^ = 0.03	Adj. R^2^ = 0.02
No depression (reference)				
Mild depression	1.59 (.91; 2.27)	.96 (.17; 1.74)	13.14 (5.59;20.68)	6.94 (-.36;14.24)
Moderate depression	.89 (.01; 1.78)	1.08 (.08; 2.08)	18.19 (8.64;27.74)	13.26 (4.13;22–38)
Severe depression	.78 (-.45; 2.02)	1.24 (-.21; 2.69)	18.45 (4.67; 32.23)	15.11 (1.90;28.32)
**Single candidate items**				
**Item 1:** Low in spirits or sad	Adj. R^2^ = 0.03	Adj. R^2^ = 0.04	Adj. R^2^ = 0.06	Adj. R^2^ = 0.03
At no time (reference)				
Some of the time	0.53 (0.24; 0.82)	0.52 (0.19; 0.84)	7.15 (3.99; 10.31)	4.88 (1.89; 7.86)
Slightly less than half the time	0.26 (-0.55; 1.08)	0.44 (-0.44; 1.33)	7.41 (-1.48; 16.31)	5.42 (-2.86; 13.71)
Slightly more than half the time	0.80 (0.08; 1.51)	0.32 (-0.52; 1.15)	16.05 (8.35; 23.75)	6.45 (-1.19; 14.09)
Most or all of the time	1.61 (0.71; 2.41)	2.32 (1.39; 3.24)	25.13 (15.81; 34.45)	19.45 (10.98; 27.93)
**Item 2:** Lost interest	Adj. R^2^ = 0.02	Adj. R^2^ = 0.03	Adj. R^2^ = 0.05	Adj. R^2^ = 0.02
At no time (reference)				
Some of the time	0.16 (-0.14; 0.47)	0.55 (0.21; 0.90)	5.25 (1.90; 8.60)	4.76 (1.63; 7.89)
Slightly less than half the time	0.98 (0.40; 1.56)	1.00 (0.36; 1.65)	16.58 (10.23; 22.93)	6.15 (0.25; 12.06)
Slightly more than half the time	0.34 (-0.23; 0.91)	0.67 (0.01; 1.33)	9.18 (3.00; 15.36)	8.81 (2.79; 14.84)
Most or all of the time	0.86 (0.17; 1.55)	0.76 (-0.01; 1.54)	13.85 (6.30; 21.40)	7.60 (0.51; 14.70)
**Item 3:** Lacking energy	Adj. R^2^ = 0.04	Adj. R^2^ = 0.05	Adj. R^2^ = 0.09	Adj. R^2^ = 0.05
At no time (reference)				
Some of the time	0.15 (-0.20; 0.49)	0.46 (0.07; 0.84)	3.92 (0.18; 7.65)	1.72 (-1.80; 5.23
Slightly less than half the time	0.67 (0.15; 1.19)	1.07 (0.49; 1.65)	12.26 (6.65; 17.88)	7.73 (2.46; 13.00)
Slightly more than half the time	0.76 (0.24; 1.28)	0.78 (0.17; 1.38)	14.34 (8.75; 19.93)	10.91 (5.37; 16.46)
Most or all of the time	1.33 (0.83; 1.82)	1.50 (0.93; 2.06)	20.71 (15.37; 26.05)	13.79 (8.64; 18.93)
**Item 4:** Felt less self-confident	Adj. R^2^ = 0.01	Adj. R^2^ = 0.02	Adj. R^2^ = 0.04	Adj. R^2^ = 0.02
At no time (reference)				
Some of the time	0.41 (0.07; 0.75)	0.38 (0.00; 0.76)	4.64 (0.92; 8.36)	4.02 (0.54; 7.50)
Slightly less than half the time	0.54 (-0.21; 1.29)	0.85 (-0.10; 1.79)	9.99 (1.88; 18.11)	9.10 (0.66; 17.54)
Slightly more than half the time	0.70 (-0.06; 1.47)	0.54 (-0.35; 1.42)	15.67 (7.41; 23.93)	7.72 (-0.35; 15.78)
Most or all of the time	1.12 (0.23; 2.02)	1.88 (0.88; 2.88)	16.83 (6.98; 26.69)	16.05 (6.91; 25.18)
**Item 8:** Restless or slowed down	Adj. R^2^ = 0.02	Adj. R^2^ = 0.005	Adj. R^2^ = 0.04	Adj. R^2^ = 0.01
At no time (reference)		*		
Some of the time	-0.03 (-0.35; 0.29)	0.27 (-0.10; 0.63)	3.76 (0.23; 7.28)	3.26 (-0.07; 6.60)
Slightly less than half the time	0.13 (-0.42; 0.69)	0.35 (-0.26; 0.95)	6.41 (0.29; 12.53)	5.42 (-0.09; 10.94)
Slightly more than half the time	0.09 (-0.45; 0.62)	0.40 (-0.21; 1.02)	5.98 (0.10; 11.86)	4.94 (-0.73; 10.60)
Most or all of the time	1.25 (0.66; 1.84)	0.86 (0.18; 1.54)	19.13 (12.67; 25.60)	8.27 (2.02; 14.53)

*All associations between items and outcomes and total MDI and outcomes were statistically significant (p < .05) except for the association between item 8 and LBP at 12-months. Confidence intervals that span across zero indicate that this particular category was not significantly different from the reference.

### Associations between the MDI total score and single items

Correlation coefficients between single items and the full MDI and the variance explained in total MDI by each item appear from Table 3. Items 2 (Have you lost interest in your daily activities?), 3 (Have you felt lacking energy and strength?), and 8 (Have you felt restless/Have you felt subdued or slowed down?) were considered candidates for brief screening questions based on the correlation coefficients, and in addition item 1 (Have you felt low in spirits or sad?) and item 4 (Have you felt less self-confident) were included in the analyses of prognostic capacity because they had R-squared values equal to that of item 8.

**Table 3:** Correlations between the total MDI score and single items and amount of variance in the MDI score explained by each item

	**Spearman’s rho**	**R-squared**
Item 1	.66	.56
Item 2	.72	.59
Item 3	.77	.62
Item 4	.63	.56
Item 5	.61	.48
Item 6	.34	.25
Item 7	.59	.42
Item 8	.74	.56
Item 9	.62	.35
Item 10	.56	.36

All correlations and regression models were statistically significant (p < .001).

### Associations between single candidate items and outcome

All candidate items were significantly associated with the outcome measures except for item 8 in relation to LBP intensity at 12-months (Table 2). Effect sizes were comparable to those of the categorised total MDI, and, as for the total scale, little of the variance in the outcomes was explained. For all outcome measures items 1 and 3 explained the most variance and were considered the best choices as brief screening questions although not performing pronouncedly better than other candidate questions.

### Prediction of outcomes by the brief screening questions as compared to the total MDI

**Figure 1:**
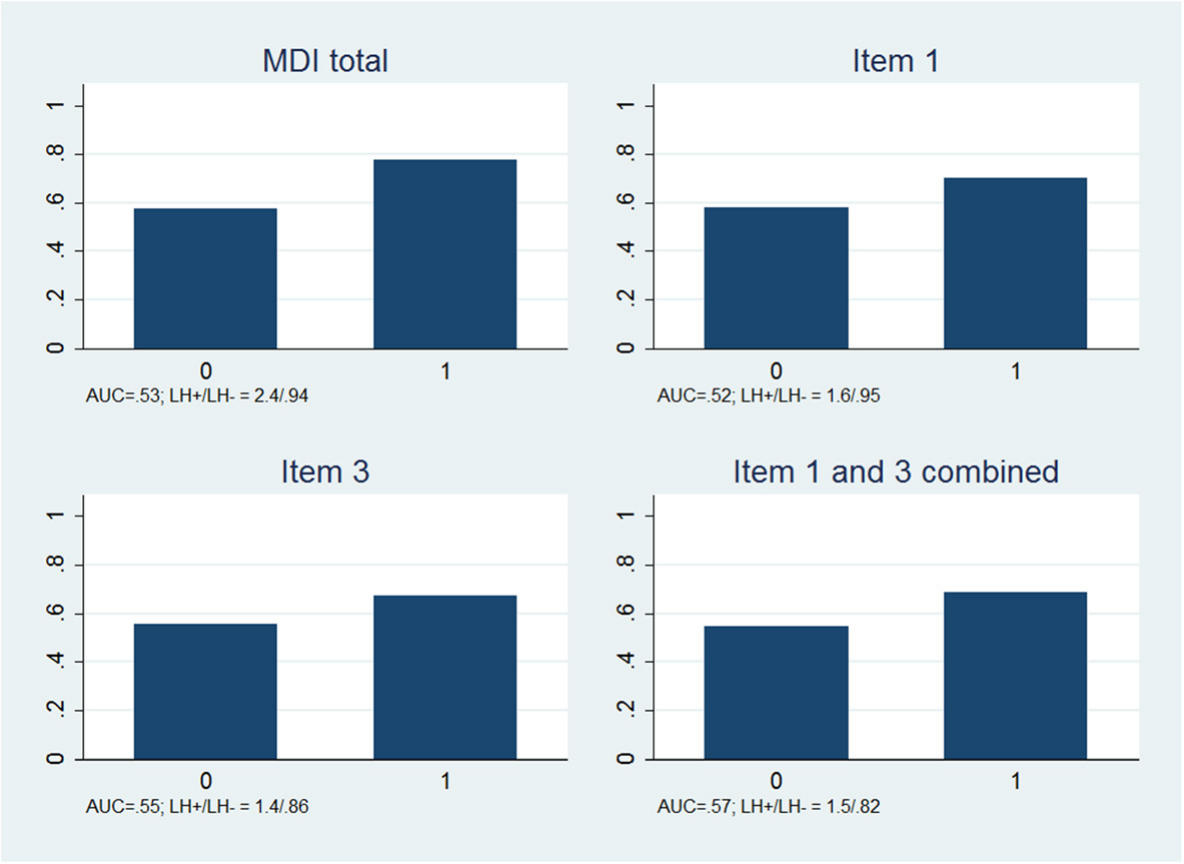
**Proportion of patients with persistent pain at 3-months follow-up in depressed (1) and non-depressed (0) according to four ways of screening.** AUC = Area under the curve. LH + = Positive likelihood ratio. LH- = Negative likelihood ratio.

Patients with signs of depression had more frequently persistent pain at 3-months follow-up regardless of whether the definition of depression was based on the total MDI, item 1, item 3, or items 1 and 3 combined (Figure 1). However, the predictive accuracy of depression was low with all definitions (AUC: 0.52 to 0.57). A slight increase in the predictive accuracy was gained by combining items 1 and 3 as compared to using only one of them. The positive likelihood ratio, i.e. the increase in risk of a poor prognosis associated with depression, was higher for the total MDI scale than for single items, whereas a combination of items 1 and 3 performed best regarding the negative likelihood ratio, i.e. identification of those with reduced risk of a poor prognosis.

**Figure 2:**
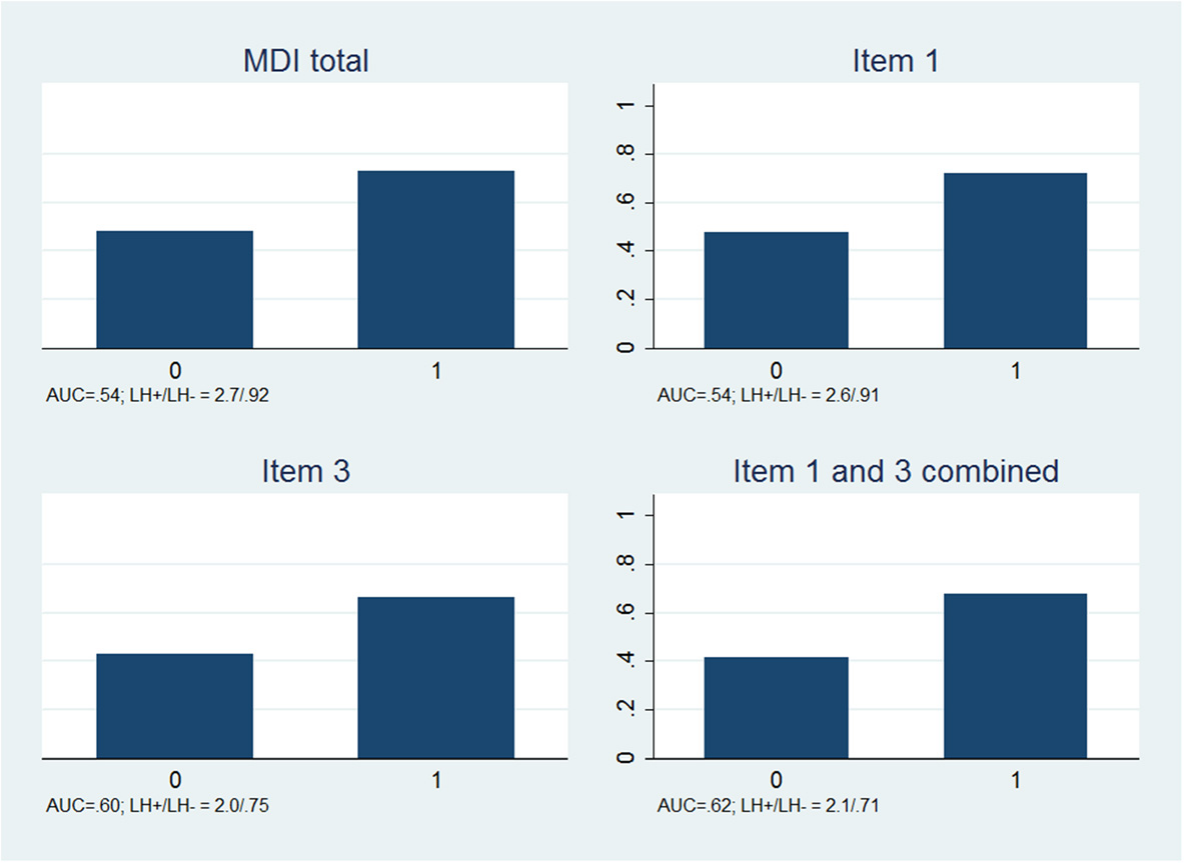
**Proportion of patients with persistent activity limitations at 3-months follow-up in depressed (1) and non-depressed (0) according to four ways of screening.** AUC = Area under the curve. LH + = Positive likelihood ratio. LH- = Negative likelihood ratio.

Depression predicted activity limitation more accurately than it predicted pain (Figure 2), but the accuracies were low, also regarding activity limitation (AUC: 0.54 to 0.62). Item 3 performed somewhat better than the total MDI score due to a lower negative likelihood ratio. Very little was achieved by combining items 1 and 3.

Predictions of 12-months outcomes were very similar to those of 3-months outcome and are not reported.

## Discussion

Our main finding was that item 1 (feeling low in spirits or sad) and item 3 (lacking energy and strength) of the MDI were reasonable alternatives to the total scale when ultra-brief screening questions are needed. In addition we found that depression measured by the MDI was associated with prognosis in patients with LBP, but prediction of outcome had low accuracy.

As in previous studies [11-13] depression was not frequent in this cohort of patients seeking chiropractic care for LBP. Therefore it was not surprising that depression on its own did not predict outcome with any certainty given that a high number of factors appear to influence the course of LBP [8]. One previous study investigating depression as a prognostic factor in chiropractic patients with LBP used the Hospital Anxiety and Depression Scale and found that depression was associated with outcome but not an independent predictor [12]. It was not within the scope of this paper to investigate whether MDI scores were independently associated with outcome. Moreover, it was not investigated if cognitive elements were included in treatment for patients with signs of depression which may reduce the predictive value of depression.

A recent study demonstrated substantial overlap between depression and pain-related psychological factors such as anxiety, self-efficacy, and kinesiophobia, and the combined construct of pain-related emotional distress was an important indicator of risk of poor prognosis in patients from general practice [31]. To examine complex psychological constructs in large clinical cohorts, simple ways of collecting information on each element are essential.

We suggest that items 1 and 3 from the MDI or the two in combination can be used as reasonable substitutes for the MDI scale, although we recognise that items 2 and 4 performed almost correspondingly. With the applied categorisation of MDI, the recommended items predicted activity limitation as well as the total MDI did and were only slightly less accurate in the prediction of pain. The accuracy in terms of AUC and the negative likelihood ratio favoured item 3 slightly over item 1, whereas item 1 should be preferred if positive predictive value is the main priority.

A 2-item screening test for depression taken from the Primary Care Evaluation of Mental Disorders Procedure (PRIME-MD) has previously been recommended for physical therapist treating LBP [15]. The PRIME-MD questions “During the past month, have you often been bothered by feeling down, depressed, or hopeless?” and “During the past month, have you often been bothered by little interest or pleasure in doing things?” reflects the same aspects of depression as MDI items 1 and 2. However, our results indicated that in the investigated setting, item 3 should be preferred to item 2. This was likely due to item 3 having high scores considerably more frequently than item 2 in our cohort. Also 1-item questions simply asking if patients feel depressed [22], or to what extent they have felt depressed during the last month [23], appear useful for diagnostic screening. However, none of these questions were tested as prognostic factors and future research in this area should provide direct comparisons of existing brief screening questions.

This study was based on a large sample from ordinary clinical practice with rather complete data available. Drop-out rates of 21% and 26% at follow-up was a limitation, and slightly higher MDI scores at baseline in those who dropped out may have resulted in underestimation of the effect of depression on outcome. However, there is no reason to believe that single items would be affected differently by drop-outs than the total scale, and we do not consider drop-out a major issue in relation to the aim of this study.

Another limitation was the categorisation of the MDI which reduced the available information but was performed since the continuous measure did not fit a linear model. We used a previously validated categorisation as this was believed to best mirror the general use of the MDI, but it is possible that other cut-points would be preferable in this population. Smoothened linear plots of outcomes as a function of the MDI sum score gave no obvious reason to alter the categorisation. It was deemed necessary to combine mild to severe depression for the calculations of the predictive capacity of the MDI which may have lowered the accuracy. However, this did not affect the choice of questions to be used for a brief screening tool.

We presented results on combining items 1 and 3 with *or* (i.e. any of the two should be positive) which increased the accuracy of prediction activity limitation slightly. In order to increase the specificity of the screening questions it would appear logical to combine the two items by *and* (i.e. both should be positive), but with the applied cut-point nothing was achieved by that.

We chose to prioritise sensitivity when dichotomising the items since we consider an increased attention on potential psychological issues in patients for whom it is irrelevant less problematic than not being aware of depressive symptoms in those for whom it matters. The cut-point had no implications for the identification of the best screening items and could be altered if wanting a lower number of false positives.

In conclusion, signs of depression were infrequent in this LBP cohort seeking chiropractic care. Depression was associated with outcome but not a major explanation of poor prognosis. Single items of the MDI performed almost as good as prognostic factors as did the entire scale and items 1 or 3 can be used as alternatives to the total scale when a brief screening tool is needed. Head-to-head comparisons with other screening questions for depression are needed in order to recommend one rather than another. In short, depression is of importance in LBP when present but not prevalent in chiropractic practice. Therefore we recommend screening for depression with a very brief tool. This could be item 1 or item 3 from the MDI.

## Competing interests

The authors have no financial or non-financial competing interests to declare. The Foundation for Chiropractic Research and Post Graduate Education, Denmark funded the study and is continuously financing the Nordic Institute of Chiropractic and Clinical Biomechanics.

## Authors’ contributions

AK formed the study idea. All authors were involved in the design of the study, interpretation of data, revision of the manuscript, and all gave final approval of the manuscript. AK performed the data analysis and wrote the initial draft of the manuscript.

## Supplementary Material

Additional file 1 Major (ICD-10) depression inventory. Click here for file
