# Supplementary figures and images for: Brief screening questions for depression in chiropractic patients with low back pain: identification of potentially useful questions and test of their predictive capacity
Source: Chiropr Man Therap. 2014 Jan 17;22:4. doi: 10.1186/2045-709X-22-4 (PMC3902415; doi:10.1186/2045-709X-22-4)

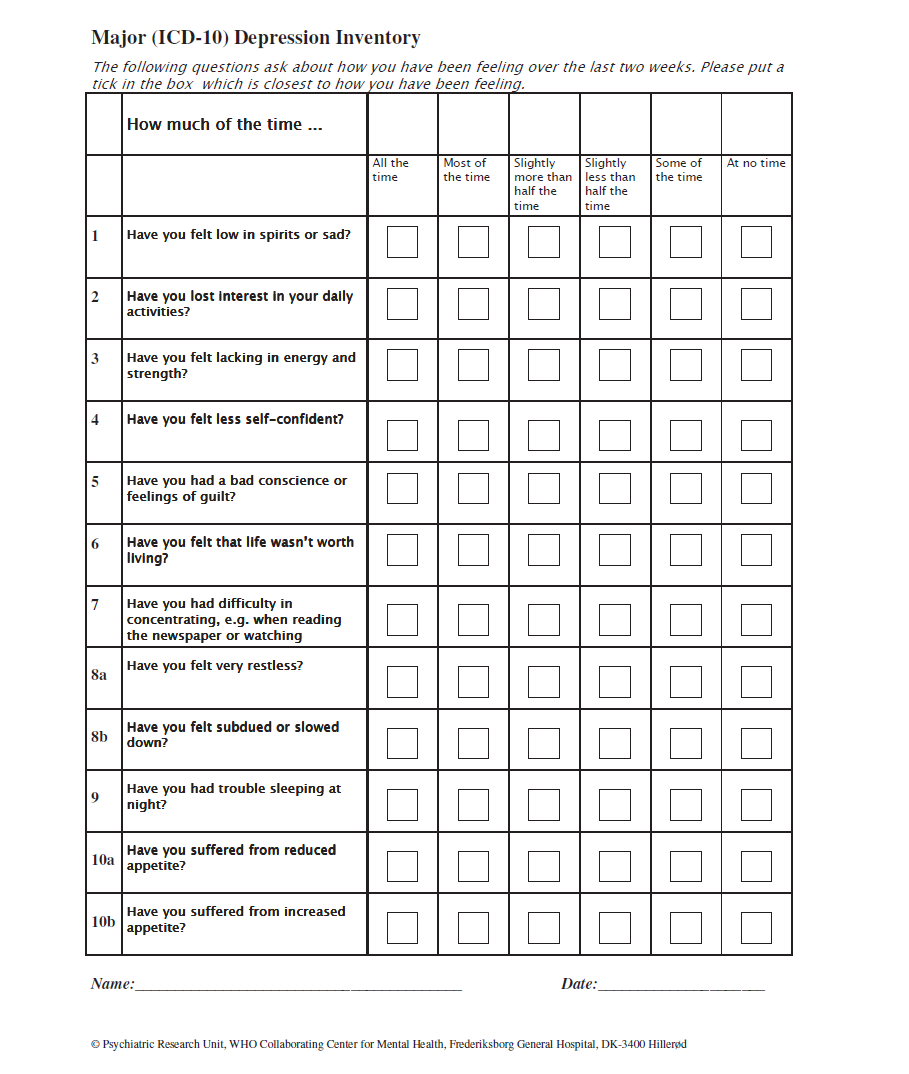

Supplement: Additional file 1 — Major (ICD-10) depression inventory. [file 2045-709X-22-4-S1.png]
